# Optimizing the Structure and Performances of Cu-MOF@Ti_3_C_2_T_X_ Hybrid Electrodes by Introducing Modulated Ligand

**DOI:** 10.3390/nano15110864

**Published:** 2025-06-04

**Authors:** Sumin Li, Xiaokun Qu, Feng Liu, Pingwei Ye, Bo Yang, Qiang Cheng, Mengkun Yang, Yijing Nie, Maiyong Zhu

**Affiliations:** 1 School of Materials Science & Engineering, Jiangsu University, Zhenjiang 212013, China; 2212205064@stmail.ujs.edu.cn (X.Q.); 2222205025@stmail.ujs.edu.cn (F.L.); cq18552374534@163.com (Q.C.); 2222305060@stmail.ujs.edu.cn (M.Y.); nieyijing@ujs.edu.cn (Y.N.); maiyongzhu@ujs.edu.cn (M.Z.); 2 State Key Laboratory of NBC Protection for Civilian, Beijing 102205, China; 12426035@zju.edu.cn

**Keywords:** 2D MOF, multiple hydrogen bonds, hybrid, supercapacitors

## Abstract

To date, two-dimensional metal–organic frameworks (2D MOFs) have attracted much attention in many fields. Owing to their ultra-high porosity and specific surface area, great structural diversity and functional tunability, as well as feasible precision design at the molecular level, 2D MOFs have won rapid development in the field of energy storage. However, as a coordination compound, MOFs possess poor structural stability and are prone to structural collapse in electrochemical reactions, which seriously limits their electrochemical performance. Therefore, there is an urgent need to improve the structural stability of MOF electrode materials. In this study, a 2D MOF@Ti_3_C_2_T_X_ hybrid was constructed, in which urea pyrimidinone isocyanate (UPy-NCO) units were introduced via a condensation reaction with the active functional groups on MOFs, thus forming multiple hydrogen bonds among MOF frameworks to strengthen their structural stability. Importantly, 2,6-diaminopyridine was utilized to modulate the structure and properties. Initially, the mono-coordination model of the N atom on a pyridine ring with metal ions could create defects and form further pores. Two −NH_2_ groups helped to improve the grafting reaction degree of UPy-NCO, leading to an increased ratio of forming quadruple hydrogen bonds (H-bonds), further strengthening the structure of the hybrid. As expected, the Cu-MOF@Ti_3_C_2_T_X_-20%DAP-UPy hybrid exhibited a specific capacitance of 148 F g^−1^ at 1 A g^−1^, which is 45% higher than that of Cu-MOF@Ti_3_C_2_T_X_-UPy (102 F g^−1^). A good capacitance retention of 88% was obtained as the current density increased from 0.2 to 5 A g^−1^. Moreover, excellent cycling stability (91.1%) was obtained at 1 A g^−1^ after 5000 cycles.

## 1. Introduction

With the rapid development of portable electronics, supercapacitors have attracted much attention due to their fast charging and discharging, high power density, green environmental protection, and long lifetime [1,2,3,4,5,6]. Due to their ultra-high porosity, large specific surface area, and structural tunability [7,8,9,10,11,12,13,14,15], MOFs have received widespread attention in supercapacitor and other energy-storage applications [16,17,18,19]. In particular, owing to their exposed active sites and higher electrical conductivity, as well as regular and well-ordered pore structure [20,21,22,23,24,25,26,27], 2D MOFs are more conducive to achieving high performance as electrode materials. However, the weak coordination bonds between metal ions and organic ligands make MOF frameworks unstable and even prone to collapse, leading to poor cycling stability [28,29,30,31,32,33,34], thus restricting their practical applications. Therefore, enhancing the cycling stability of MOF energy-storage materials needs to be addressed urgently.

Self-healing ability provides a novel design idea for improving the service life of energy storage devices. Currently, two self-healing categories are mainly investigated: externally assisted self-healing and intrinsic self-healing [35,36,37]. Externally assisted self-healing is realized by incorporating self-healing agents into the materials [38,39,40]. For example, Guo [41] et al. investigated epoxy matrix microencapsulated self-healing materials. However, most microcapsule-based self-repairs exhibit only onetime self-repair properties, which severely limits their practical applications. Intrinsic self-healing materials can repair by themselves through the introduction of reversible chemical bonds [37,41,42,43,44,45] (e.g., boronic acid diester bonds, hydrogen bonds, etc.). For example, in 2023, Wang [45] et al. sprayed hydrophobically modified CNT and NiO/CoO nanoparticles onto the surface of a hydrogel (PVA) to construct electrodes, which utilized the dynamic hydrogen bonds formed between PVA and HCl through physical cross-linking to provide the self-healing ability. The as-prepared supercapacitor achieved a capacitance retention of 75.4% after 2000 cycles. Both the introduction of self-healing agents and reversible chemical bonds can give energy storage devices self-healing abilities, which is important for prolonging their service life [46,47,48,49,50,51]. However, traditional self-healing materials are usually polymers, which are not electrochemically active and thus unfavorable for producing capacitance, leading to the decreased electrochemical performance of electrodes or energy storage devices [52,53,54,55,56,57,58].

We have proposed an innovative strategy to enhance the structural stability of MOFs by introducing quadruple hydrogen bonds directly into MOF frameworks [23]. Here, to further enhance the structural stability and performance of MOF-based electrodes, a modulated linker, 2,6-diaminopyridine, was utilized. On the one hand, the mono-coordination model of the N atom on a pyridine ring with metal ions could produce defects and form further pores. On the other hand, the two −NH_2_ groups help to improve the grafting reaction degree of UPy-NCO units, improving the ratio of forming quadruple hydrogen bonds, further strengthening their structure stability.

## 2. Materials and Methods

### 2.1. Preparation of Samples

The materials, reagents, and synthesis of Ti_3_C_2_T_X_, copper oxide nanosheets (Cu-ONS), Cu-MOF@Ti_3_C_2_T_X_, and UPy-NCO are displayed in the Supporting Information (SI).

Synthesis of Cu-MOF@Ti_3_C_2_T_X_-DAP: H_2_BDC-NH_2_ (30 mg), 2,6-diaminopyridine (0, 3, 6, 9 mg), and PVP (50 mg) were dissolved in a mixed solvent containing 1.1 mL of DMF, 1.1 mL of deionized (DI) water, and 1.1 mL of ethanol, obtaining a mixed solution. Cu-ONS (25 mg) was dispersed in 5 mL of DI water under stirring, and then Ti_3_C_2_T_X_ (10 mg) was added, forming another solution. Then, the two solutions were together transferred to a reactor and remained at 100 °C for 20 h. Lastly, the sample was washed and dried, and denoted as Cu-MOF@Ti_3_C_2_T_X_-X%DAP (X = 10, 20 and 30).

**Scheme 1:**
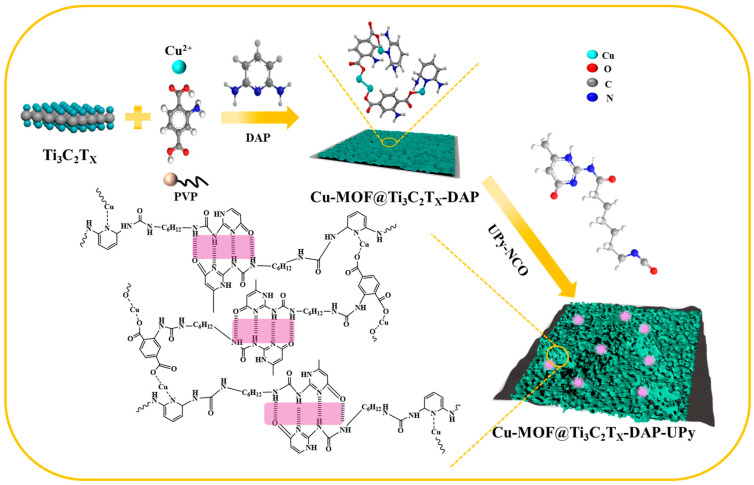
Schematic illustration of Cu-MOF@Ti_3_C_2_T_X_-DAP-UPy.

Synthesis of Cu-MOF@Ti_3_C_2_T_X_-DAP-UPy: 40 mg of Cu-MOF@Ti_3_C_2_T_X_-DAP was dissolved in 20 mL of DMF and 30 mg of UPy-NCO was dissolved in 10 mL of DMF. Then, the two solutions were mixed, followed by the addition of 2 drops of dibutyltin dilaurate. The mixed solution was maintained at 90 °C for 20 h under stirring. Finally, the sample was washed with ethanol, dried at 40 °C, and denoted as Cu-MOF@Ti_3_C_2_T_X_-DAP-UPy, as described in Scheme 1.

### 2.2. Electrochemical Characterization

The working electrode material consisted of acetylene black, polyvinylidene chloride (PVDF) and active material. The three materials were mixed (mass ratio of 8:1:1) with N-methylpyrrolidone to form a homogeneous paste. Then, the paste was coated on a piece of Ni foam (1 × 1 cm^2^) current collector, followed by a drying process at 80 °C for 12 h, thus obtaining a working electrode (the loading amount was 1.5~2 mg cm^−2^).

An electrochemical workstation (CHI760E) was used to perform electrochemical experiments including cyclic voltammetry (CV), constant current charge–discharge (GCD) and electrochemical impedance spectroscopy (EIS). A three-electrode system was used to investigate the performance of single electrodes, in which the working electrode, counter electrode, and reference electrode were the as-prepared material, Pt plate, and Hg/HgO, respectively. A 1 M KOH aqueous solution was used as the electrolyte. The capacitance (C, F g^−1^) values were evaluated by calculating the integral of the area under the charging and discharging part of the curves as follows [48]:

(1) C=I∫(1/m×V(t))dt=IΔt/mΔV

where *I* (A g^−1^) is the current density, Δ *t* (s) is the discharge time, *m* (g) is the mass of the active material, and *ΔV* (V) is the potential.

In addition, the energy density (*E*, Wh kg^−1^), power density (*P*, W kg^−1^) and Coulombic Efficiency (CE) of the prepared device was investigated using the following formulae [56]:

(2) $$E=\frac{C\times {∆V}^{2}}{2\times 3.6}$$

(3) $$P=\frac{3600\times E}{∆t}$$

(4) $$CE=\frac{I_{d}t_{d}}{I_{c}t_{c}}\times 100\%$$

where Δ*t* (s) is the discharge time, Δ*V* (V) is the potential window, *I_d_* (A) is the discharge current, *t_d_* (s) is the discharge time, *I_c_* (A) is the charge current, and *t_c_* (s) is the charge time.

## 3. Results

### 3.1. Structural Characterization

**Figure 1:**
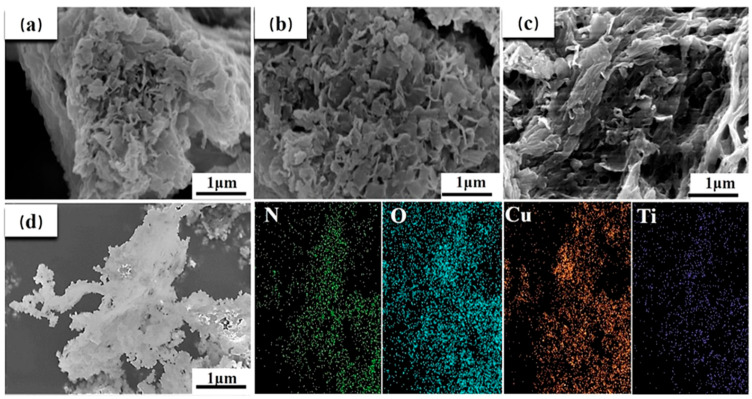
SEM images of the samples. (**a**) Cu-MOF@Ti_3_C_2_T_X_-10%DAP-UPy, (**b**) Cu-MOF@Ti_3_C_2_T_X_- 20%DAP-UPy, (**c**) Cu-MOF@Ti_3_C_2_T_X_-30%DAP-UPy, and (**d**) EDS images of Cu-MOF@Ti_3_C_2_T_X_-20%DAP-UPy with the corresponding elemental distributions of N, O, Cu and Ti.

The SEM images of the samples are shown in Figure 1 and Figure S1. As displayed in Figure S1a, lots of nanosheets with a size of about 1 μm were observed in Cu-MOF@Ti_3_C_2_T_X_. With the addition of 2,6-diaminopyridine, the microstructure of the samples changed obviously. The size of the lamellar structure became smaller with the increasing 2,6-diaminopyridine content. The mono-coordination model of the N atom on the pyridine ring with metal ions created defects and formed further pores, leading to the decrease in size of the nanosheets (Figure S1b,c). For Cu-MOF@Ti_3_C_2_T_X_-30%DAP, lots of irregular fragments can be seen (Figure S1d), caused by the large number of defects in the nanosheets due to the excess of 2,6-diaminopyridine [59]. After the UPy-NCO units were grafted via a condensation reaction with the −NH_2_ active groups on the MOFs, the morphology of the Cu-MOF@Ti_3_C_2_T_X_-DAP-UPy samples (Figure 1) were further changed. Especially, the Cu-MOF@Ti_3_C_2_T_X_-30%DAP-UPy (Figure 1c) exhibited a completely different morphology compared with Cu-MOF@Ti_3_C_2_T_X_-30%DAP (Figure S1d), showing a thinner layer and cross-linked network structure. As the amount of 2,6-diaminopyridine increased up to 30%, more −NH_2_ active groups were introduced onto the MOFs, leading to an improvement in the grafting reaction degree of UPy-NCO and a further increase in the ratio of forming quadruple hydrogen bonding, subsequently promoting the formation of a cross-linked network structure. The EDS elemental analysis (Figure 1d) shows that the elements, N, O, Cu, and Ti, are uniformly distributed in Cu-MOF@Ti_3_C_2_T_X_-20%DAP-UPy sample.

**Figure 2:**
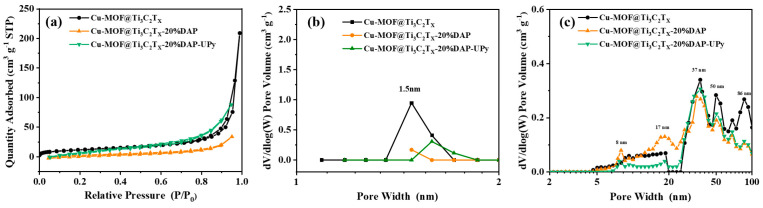
(**a**) N_2_ adsorption–desorption isotherms, (**b**) DFT pore-size distribution curves and (**c**) BJH pore-size distribution curves of the samples.

The N_2_ adsorption–desorption tests were performed to investigate the pore characteristics of the prepared materials, as shown in Figure 2. All the samples exhibit type IV curves (Figure 2a), indicating the co-existence of micropores and mesopores [60]. The hierarchical pores, including micropores (1.5 nm), mesopores (8, 17, 37 and 50 nm), and macropores (50–86 nm), are clearly displayed in Figure 2b,c. Most importantly, an 8 nm mesopore can be noticed in both Cu-MOF@Ti_3_C_2_T_X_-20%DAP and Cu-MOF@Ti_3_C_2_T_X_-20%DAP-UPy, which might be due to the defect originating from the mono-coordination model between 2,6-diaminopyridine and Cu^2+^. Moreover, the specific surface areas of Cu-MOF@Ti_3_C_2_T_X_, Cu-MOF@Ti_3_C_2_T_X_-20%DAP, and Cu-MOF@Ti_3_C_2_T_X_-20%DAP-UPy are 43.1, 27.4, and 14.6 m^2^ g^−1^, respectively. As a modulated linker, 2,6-diaminopyridine participated in the growth of MOFs, as well as the grafting reactions with UPy-NCO units, resulting in a decrease in the proportion of 1.6 nm micropores, thus causing a decrease in the specific surface area.

The XRD patterns of the samples are displayed in Figure S2. Compared with Cu-MOF@Ti_3_C_2_T_X_-UPy, the samples containing 2,6-diaminopyridine exhibit new diffraction peaks at 2θ of 5.9°, 11.9°, 25.3°, 26.8°, and 43.2°, which is due to the formation of new crystal structures stemming from the coordinate reactions between Cu^2+^ and N atoms on the pyridine rings [61].

**Figure 3:**
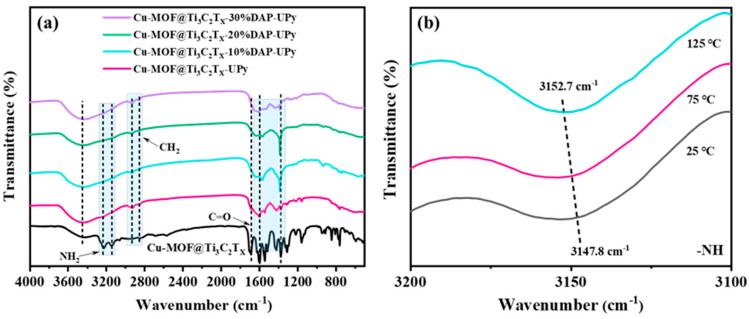
(**a**) FT−IR spectra of samples and (**b**) temperature-variable FT-IR spectra of Cu-MOF@Ti_3_C_2_T_X_-20%DAP-UPy.

Figure 3a shows the FT-IR spectra of the samples. For Cu-MOF@Ti_3_C_2_T_X_, the two peaks at 3237 and 3142 cm^−1^ are ascribed to the −NH_2_ stretching vibrations [62], while the strong and sharp characteristic peak at 1689 cm^−1^ is caused by the C=O stretching vibrations. For the samples grafted with UPy-NCO, several obvious changes can be noticed in the peaks. First, the broad peak at 3454 cm^−1^ associated with the −OH is strengthened in intensity, which is attributed to the formation of intermolecular H-bonds (C=O···H−N). Second, the characteristic peaks at 2936 and 2861 cm^−1^ can be noticed, which are caused by the −CH_2_ stretching vibrations, implying that the UPy-NCO units were successfully grafted onto the MOFs. Most importantly, the characteristic peak corresponding to the C=O stretching vibrations exhibited a redshift in wavenumber and presented a blunt shape in profile, which is also associated with the intermolecular H−bonds (C=O···H−N). As the content of 2,6-diaminopyridine increased, the characteristic peak of C=O gradually became broader, proving that more hydrogen bonds were formed. Particularly, for Cu-MOF@Ti_3_C_2_T_X_-30%DAP-UPy a broad adsorption band at around 1640–1380 cm^−1^ was formed, caused by the peak shift of C=O, as well as the peak overlap of different groups including C=C and C=N. It is worth stressing that the −NH bonds are also an important component taking part in the formation of quadruple hydrogen bonds, which can be further identified by the variable FT-IR spectra (Figure 3b). When the temperature was increased from 25 °C to 125 °C, with the dissociation of hydrogen bonds, the peak associated with the stretching vibration of the −NH bonds shifted slightly from 3147.8 cm^−1^ to 3152.7 cm^−1^ [63,64].

**Figure 4:**
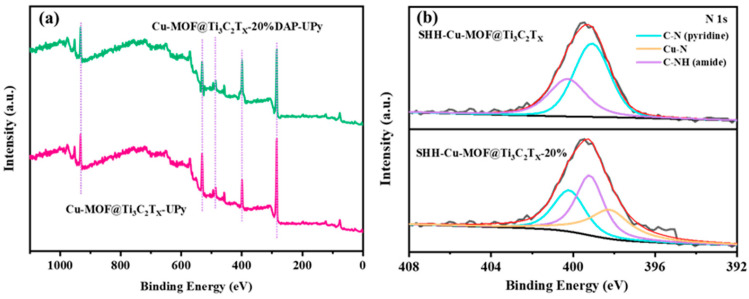
XPS spectra of samples: (**a**) survey spectrum, (**b**) high-resolution XPS spectra of N 1s.

Figure 4 shows the XPS spectra of Cu-MOF@Ti_3_C_2_T_X_ and Cu-MOF@Ti_3_C_2_T_X_-20%DAP-UPy. As displayed in Figure 4a, with the introduction of 2,6-diaminopyridine, the peak intensity of the N element in Cu-MOF@Ti_3_C_2_T_X_-20%DAP-UPy is highlighted compared to that of Cu-MOF@Ti_3_C_2_T_X_, which is attributed to the increase in grafting density of the UPy-NCO units. Moreover, in the N 1s spectra of Cu-MOF@Ti_3_C_2_T_X_-20%DAP-UPy (Figure 4b), the peak corresponding to the Cu−N (399.3–399.7 eV) can be observed, further confirming the coordinate reaction between the N atoms on the pyridine rings and Cu^2+^ ions. The other two peaks are assigned to C−N on pyridine (400.3–400.7 eV), and C−N formed on the amide bond (398.3–399 eV) [65].

### 3.2. Electrochemical Performance

**Figure 5:**
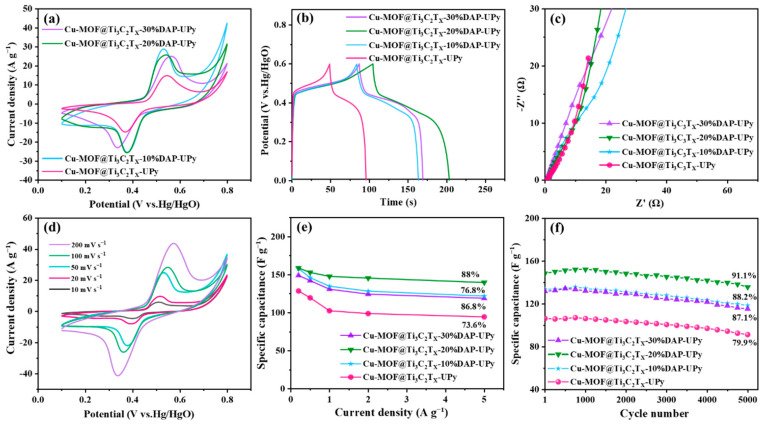
Electrochemical performances of samples: (**a**) CV profiles at 50 mV s^−1^, (**b**) GCD profiles at 1 A g^−1^, (**c**) Nyquist plots, (**d**) CV profiles of Cu-MOF@Ti_3_C_2_T_X_-20%DAP-UPy, (**e**) specific capacitances at various current densities, and (**f**) cycling performances at 1 A g^−1^.

The electrochemical properties of the prepared samples are shown in Figure 5. The CV curves of different samples at a scan rate of 50 mV s^−1^ are shown in (Figure 5a). Obvious redox peaks can be observed in CV curves, which stems from the redox reactions of Cu−O clusters in the Cu-MOF as follows:

(5) Cu−O + OH^−^ ↔ CuOOH + e^−^

Moreover, the introduction of Ti_3_C_2_T_X_ could also produce pseudo-capacitance [66,67]. The combination with non-ideal triangles in the GCD curves (Figure 5b) suggests the two electrochemical behaviors coexist: pseudo-capacitance and double-layer capacitance. Moreover, the longer discharging times indicate that the samples with the addition of 2,6-diaminopyridine exhibit higher capacitances compared with Cu-MOF@Ti_3_C_2_T_X_-UPy. As mentioned above, the mono-coordination model of the N atom on the pyridine ring with Cu^2+^ ions could create defects and form further pores, exposing more active sites and facilitating redox reactions, leading to enhanced capacitances. However, abundant defects and pores prolong the ion transportation path and reduce the ion diffusion rate, which can be demonstrated by the lower slope of the straight lines in the low-frequency region (Figure 5c). Notably, the Cu-MOF@Ti_3_C_2_T_X_-20%DAP-UPy exhibits the best electrochemical performance, delivering a specific capacitance of 148 F g^−1^ at 1 A g^−1^. With the increased scan rates (Figure 5d), similar shapes of CV curves were maintained, indicating good reversibility.

**Figure 6:**
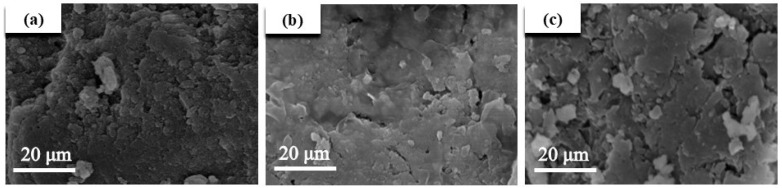
SEM images of (**a**) Cu-MOF@Ti_3_C_2_T_X_-10%DAP-UPy, (**b**) Cu-MOF@Ti_3_C_2_T_X_-20%DAP-UPy, and (**c**) Cu-MOF@Ti_3_C_2_T_X_-30%DAP-UPy electrodes after cycling tests.

The calculated capacitances investigating the effect of 2,6-diaminopyridine on the performances of the samples are shown in Figure 5e. With the current density increased from 0.2 to 5 A g^−1^, the capacitance retentions of Cu-MOF@Ti_3_C_2_T_X_-UPy, Cu-MOF@Ti_3_C_2_T_X_-10%DAP-UPy, Cu-MOF@Ti_3_C_2_T_X_-20%DAP-UPy, and Cu-MOF@Ti_3_C_2_T_X_-30%DAP-UPy are 73.6%, 76.8%, 88%, and 86.8%, respectively. Furthermore, the cycling performances were investigated, as shown in Figure 5f. After 5000 cycles, the capacitance retentions of the above samples are 79.9%, 88.2%, 91.1% and 87.1%, respectively. The participation of 2,6-diaminopyridine not only improves the storage capacitance of the samples, but also optimizes their rate performance and cycle stability. The −NH_2_ group on 2,6-diaminopyridine helps to facilitate the grafting reaction of UPy-NCO, and promotes the formation of quadruple hydrogen bonding, thus strengthening the structural stability of the hybrid. However, excessive 2,6-diaminopyridine accordingly produces more defects, weakening the crystal structure of the MOF; thus, the Cu-MOF@Ti_3_C_2_T_X_-30%DAP-UPy shows a decrease in the rate and cycle performances. As shown in Figure 6, compared with the other two samples, the Cu-MOF@Ti_3_C_2_T_X_-20%DAP-UPy presents a relatively complete surface structure. The Cu-MOF@Ti_3_C_2_T_X_-20%DAP-UPy exhibits a high capacitance retention of 91.1% after 5000 cycles, which is superior to some other MOF-based electrodes in the literature (e.g., Table 1).

The kinetic mechanism of the as-obtained hybrid was investigated by using the b-value model as follows [69]:

(6) *i* = a*ν*^b^

where *i* is the peak current and *v* is the scan rate. Especially, *b* is an adjustable parameter and has two critical values of 0.5 and 1.0, referring to the diffusive-controlled behavior and surface capacitive-controlled behavior, respectively.

**Figure 7:**
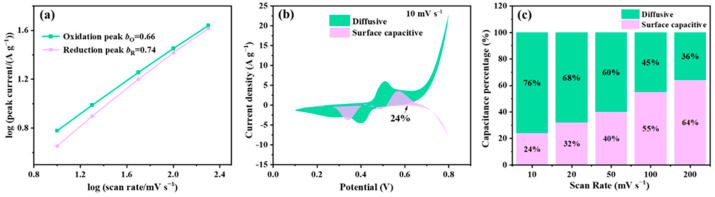
(**a**) Plots of log(i) against log(ν), (**b**) surface capacitive and diffusive contributions at scan rate of 10 mV s^−1^, and (**c**) capacitance contribution ratios.

In addition, to reveal the energy-storage mechanism in detail the Dunn’s model was also used, in which the contributions of diffusive-controlled behavior and surface capacitive-controlled behavior are descripted as follows [70]:

(7) *i(V)* = k_1_*ν* + k_2_*ν*^0.5^

where k_1_*ν* and k_2_*ν*^0.5^ correspond to the capacitive and diffusion-controlled currents, respectively. As shown in Figure 7a, for the Cu-MOF@Ti_3_C_2_T_X_-20%DAP-UPy, the calculated *b* values of the oxidation and reduction peaks are 0.66 and 0.74, respectively, indicating that the charge storage process includes both diffusive-controlled behavior and surface capacitive behavior. Combining the calculated contributions of the two charge storage behaviors (Figure 7b,c), it is obvious that the diffusive contribution is superior to the surface capacitive contribution even at 50 mV s^−1^. The formation of defects derived from the coordination reaction between 2,6-diaminopyridine and Cu^2+^ ions could promote ions to thoroughly penetrate materials, making full use of active sites to produce more capacitance. With the increased scan rates, the ions cannot diffuse sufficiently inside active materials in time, thus the diffusive-controlled proportion decreased while the surface capacitive contribution increased.

### 3.3. Electrochemical Properties of ASC Device

An asymmetric supercapacitor (Cu-MOF@Ti_3_C_2_T_X_-20%DAP-UPy//AC) was assembled using Cu-MOF@Ti_3_C_2_T_X_-20%DAP-UPy as the positive electrode, activated carbon (AC) (Figure S3) as the negative electrode, and 1 M KOH aqueous solution as the electrolyte, respectively.

**Figure 8:**
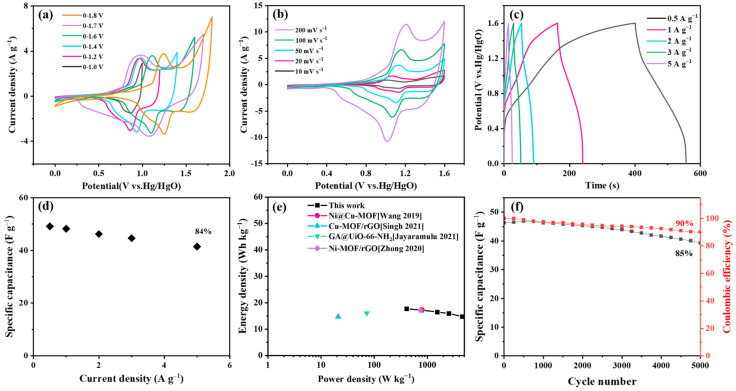
Electrochemical performance of Cu-MOF@Ti_3_C_2_T_X_-20%DAP-UPy//AC device. (**a**) CV profiles at various voltage windows at scan rate of 50 mV s^−1^, (**b**) CV profiles at various scan rates, (**c**) GCD curves under different current densities, (**d**) specific capacitances at different current densities, (**e**) Ragone plots [72,73,74,75], (**f**) cycling stability and coulombic efficiency at current density of 2 A g^−1^.

The CV curves of the device under different voltage windows from 0–1.0 to 0–1.8 V are displayed in Figure 8a. When the voltage window was set as 1.7–1.8 V, a slight polarization phenomenon can be observed, thus the 0–6 V was chosen as the appropriate working voltage. The CV curves at different scan rates are shown in Figure 8b, exhibiting similar shapes without obvious deformation, indicating that the device possesses good capacitive behavior even under fast charging/discharging. Based on the GCD curves of the device (Figure 8c), the specific capacitances were calculated and are displayed in Figure 8d. At 0.5 A g^−1^, the device delivers a specific capacitance of 50 F g^−1^, exhibiting a capacitance retention of 85% as the current density was increased to 5 A g^−1^. A maximum energy density of 17.9 W h kg^−1^ was obtained (Figure 8e), which is superior to that of some reported MOF-based devices [64,74,75,76,77,78]. The cycle stability of the device is shown in Figure 8f: a capacitance retention of 85% was delivered after 5000 cycles at 2 A g^−1^. Moreover, the calculated coulombic efficiency was 90%.

## 4. Discussion

To enhance the structural stability of MOF-based electrodes, we have explored a strategy of introducing quadruple hydrogen bonds onto MOFs. Here, to further promote the introduction of quadruple hydrogen bonds and optimize the performance of 2D MOF electrodes, 2,6-diaminopyridine was used as a modulated linker to participate in the growth of MOF and the grafting reaction of UPy-NCO. Due to the mono-coordination reaction between the pyridine ring and Cu^2+^ ions, new mesopores (8–20 nm) were introduced, enriching the pore architecture of the MOF@Ti_3_C_2_T_X_ hybrid. Profiting from the enhanced grafting reaction between the −NH_2_ groups and UPy-NCO units, quadruple hydrogen bonds among frameworks were increased, leading to the formation of hierarchical cross-linked networks. The as-obtained Cu-MOF@Ti_3_C_2_T_X_-20%DAP-UPy presented a capacitance of 158 F g^−1^ at 0.2 A g^−1^, delivering a capacitance retention of 88% at 5 A g^−1^, while the Cu-MOF@Ti_3_C_2_T_X_-UPy exhibited a capacitance of 128 F g^−1^ at 0.2 A g^−1^, presenting a 73.6% retention at 5 A g^−1^. Furthermore, the Cu-MOF@Ti_3_C_2_T_X_-20%DAP-UPy delivered a 91.1% capacitance retention after 5000 cycles, which is much superior to that of Cu-MOF@Ti_3_C_2_T_X_-UPy (79.9%), presenting an apparent increase in cycling performance. The prepared ASC device presented a specific capacitance of 50 F g^−1^ at 0.2 A g^−1^, an energy density of 17.9 Wh kg^−1^, and a capacitance retention of 85% after 5000 cycles at 2 A g^−1^. Our strategy not only provides ideas for designing novel and efficient MOFs, but also provides innovative ideas for exploring novel porous frameworks with self-healing ability.

## 5. Conclusions

In this study, the structural stability and electrochemical performance of two-dimensional MOF electrodes were optimized by introducing a quadruple hydrogen bonding strategy; 2,6-diaminopyridine (DAP) was employed as a moderating ligand involved in MOF growth, and the quadruple hydrogen bonding network was strengthened by the grafting reaction of its amino group with UPy-NCO. It was found that the mono-coordination of the pyridine ring with Cu^2^⁺ formed 8–20 nm new mesopores, which enriched the pore structure of MOF@Ti_3_C_2_T_X_ composites; the modified material, Cu-MOF@Ti_3_C_2_T_X_-20% DAP-UPy, reached a specific capacity of 15.5 mm at 0.2 A g^−1^ with a specific capacity of 158 F g^−1^ (23% enhancement), 88% capacity retention at 5 A g^−1^ (control 73.6%), and 91.1% capacity retention after 5000 cycles (control 79.9%); and the assembled asymmetric supercapacitor (ASC) had an energy density of 17.9 Wh kg^−1^ and 85% capacity retention after 5000 cycles. This strategy not only improves the conductivity and cycling stability of MOF electrodes by constructing a hierarchical cross-linking network, but also provides an innovative idea for the design of porous framework materials with a self-healing function.

## Figures and Tables

**Table 1:** Cycle stability of MOF-based electrode materials.

Electrode Materials	Electrolyte	Cyclic Stability	Ref.
Ni_3_(HITP)_2_	0.5 M Na_2_SO_4_	84% (after 10,000 cycles 0.1 mA cm^−2^)	[68]
Cu-CAT NWAs	3 M KCl	80% (after 5000 cycles 0.5 A g^−1^)	[69]
Ni/Co-BTC (2:1)	6 M KOH	60% (after 3000 cycles 5.0 A g^−1^)	[70]
NiCo-MOF	2 M KOH	76.3% (after 5000 cycles 5.0 A g^−1^)	[71]
Ni@Cu-MOF	6 M KOH	63% (after 5000 cycles 1.0 A g^−1^)	[72]
Cu-MOF/rGO	1 M Na_2_SO_4_	83.3% (after 4000 cycles 10 A g^−1^)	[73]
Cu-MOF@Ti_3_C_2_T_X_-20%DAP-UPy	1 M KOH	91.1% (after 5000 cycles 1.0 A g^−1^)	This work

## Data Availability

The data presented in the study are available on request from the corresponding authors.

## Supplementary Materials

The following supporting information can be downloaded at: https://www.mdpi.com/article/10.3390/nano15110864/s1, Figure S1. SEM images of the samples. Figure S2. XRD patterns of the samples. Figure S3. (a) CV curves of AC and Cu-MOF@Ti_3_C_2_T_X_-20%DAP-UPy at 50 mV s^−1^ and (b) GCD curve of AC at 1 A g^−1^.
