# Supplementary material for: Optimizing the Structure and Performances of Cu-MOF@Ti3C2TX Hybrid Electrodes by Introducing Modulated Ligand
Source: Nanomaterials (Basel). 2025 Jun 4;15(11):864. doi: 10.3390/nano15110864 (PMC12156962; doi:10.3390/nano15110864)
Supplement: Supplementary file 1 [file nanomaterials-15-00864-s001.zip › nanomaterials-3656162-supplementary.pdf]

Support Information
Optimizing the structure and performances of Cu-MOF@$Ti_3C_2T_X$ hybrid electrodes by introducing modulated ligand

Sumin Li [1,*], Xiaokun Qu [1], Feng Liu[1,2], Pingwei Ye [2,*], Bo Yang [2], Qiang Cheng [1], Mengkun Yang [1], Yijing Nie [1], Maiyong Zhu [1]

[1] *School of Materials Science & Engineering, Jiangsu University, Zhenjiang 212013, China*

[2] *State Key Laboratory of NBC Protection for Civilian, Beijing 102205, China*

[*] *Corresponding authors. E-mail addresses:* li_sm@ujs.edu.cn (S. Li), yepw2001@163.com (P. Ye)

Experimental

Materials and characterization

Reagents. All chemicals in the experiments were not further purified. N,N-dimethylformamide (DMF), anhydrous ethanol, acetone, concentrated hydrochloric acid (HCl), lithium fluoride (LiF), 2-aminoterephthalic acid ($H_2BDC-NH_2$), 2,6-diaminopyridine (DAP), aluminum titanocarbonate (Ti_3AlC_2), copper nitrate trihydrate ($Cu(NO_3)_2 \cdot 3H_2O$), polyvinylpyrrolidone K-30 (PVP), 2-Amino-4-hydroxy-6-methylpyrimidine (UPy) and hexamethylene diisocyanate (HDI). All chemicals in the experiment were not further purified.

Synthesis of $Ti_3C_2T_X$.

20 ml of 9 M HCI and 1.6 g of LiF were mixed to obtain a solution. Then 1 g of aluminum carbon titanate was added, and kept at 40 ˚C for 48 h. The collected product was washed with DI water for several times until the pH of the supernatant was close to 6. Then, 40 ml of deionized water was added, and kept sonicating for 1 h in an ice bath. Further, the solution was centrifuged at 3500 r for 15 min to collect the upper suspension, followed by drying at 80 ℃ for 12 h.

Synthesis of copper oxide nanosheets (Cu-ONS).

0.482 g (2 mmol) $Cu(NO_3)_2 \cdot 3H_2O$ and 0.2 g $NaBH_4$ were dissolved in 50 ml and 20 ml deionized water, respectively. Then, the two solutions were mixed and stirred for 10 min. The as-obtained product was washed with ethanol for 3 times and then dried at 60 ℃ for 48 h.

Synthesis of Cu−MOF@$Ti_3C_2T_X$.

25 mg of Cu-ONS and 10 mg of $Ti_3C_2T_X$ were dispersed in 6.7 mL of deionized (DI) water

under stirring, followed by sonication for 1 h to form a mixed solution. In parallel, 30 mg of $H_2BDC-NH_2$ and 50 mg of PVP were dissolved in a mixed solvent consisting of DMF (1.1 mL), DI water (1.1 mL) and ethanol (1.1 mL), obtaining another solution. The two solutions were then transferred into an autoclave and kept at 100 °C for 20 h. Finally, the product was washed and dried, obtaining Cu-MOF@$Ti_3C_2T_X$.

Synthesis of UPy-NCO.

UPy (6 mmol, 0.751 g) was mixed with HDI (42 mmol, 7.064 g). The mixture was then placed in a flask and stirred for 15 h at 100 °C in an oil bath (in N_2). The product was washed three times with hexanoyl combustion and then dried under vacuum at 60 °C for 12 h.

Characterization

Scanning electron microscopy (SEM) was used to analyze the surface morphology of samples. Fourier transform infrared (FTIR), X-ray diffraction (XRD) and X-ray photoelectron spectroscopy (XPS) were performed to investigate the elemental composition of materials. The specific surface area and pore characteristic of samples were analyzed via a physisorption analyzer.

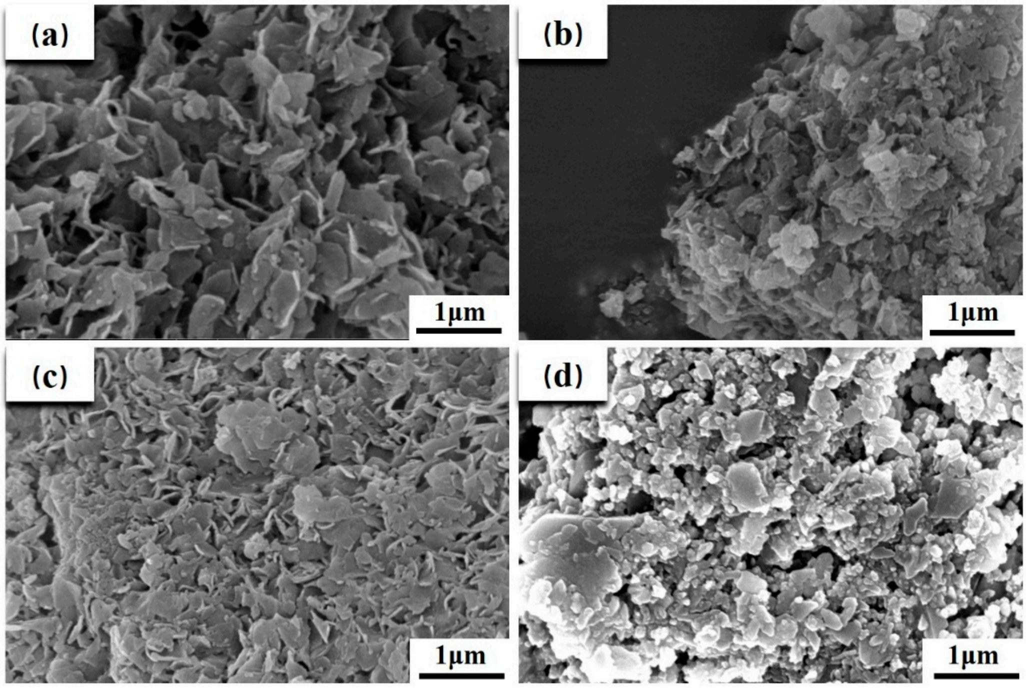

Figure S1. SEM images of the samples. (a) Cu-MOF@$Ti_3C_2T_X$, (b) Cu-MOF@$Ti_3C_2T_X$-10%DAP, (c) Cu-MOF@$Ti_3C_2T_X$-20%DAP, (d) Cu-MOF@$Ti_3C_2T_X$-30%DAP.

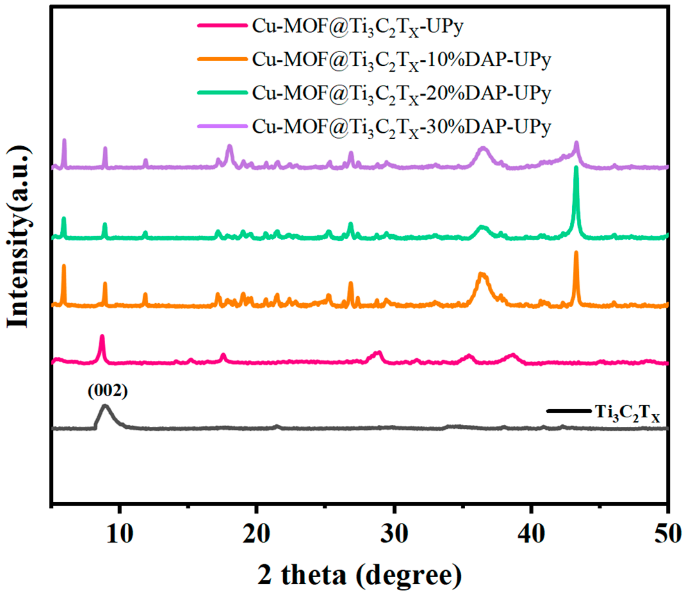

Figure S2. XRD patterns of the samples.

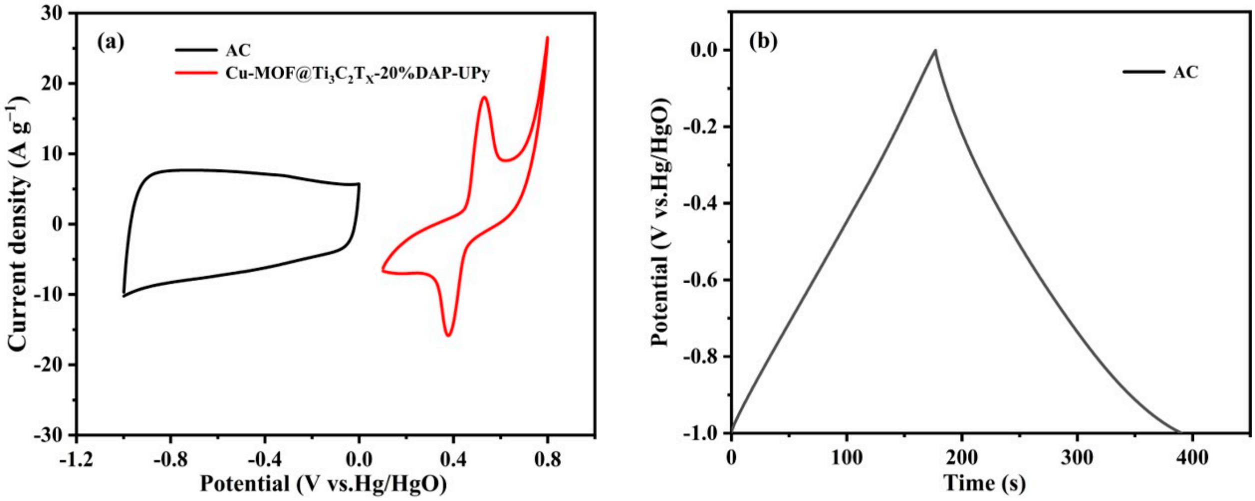

Figure S3. (a) CV curves of AC and Cu-MOF@$Ti_3C_2T_X$-20%DAP-UPy at 50 mV s^{-1} and (b) GCD curve of AC at 1 A g^{-1}.
